# Grain legume yields are as stable as other spring crops in long-term experiments across northern Europe

**DOI:** 10.1007/s13593-018-0541-3

**Published:** 2018-11-02

**Authors:** Moritz Reckling, Thomas F. Döring, Göran Bergkvist, Frederick L. Stoddard, Christine A. Watson, Sylvia Seddig, Frank-M. Chmielewski, Johann Bachinger

**Affiliations:** 1 Leibniz Centre for Agricultural Landscape Research, Eberswalder Str. 84, 15374 Müncheberg, Germany; 2 Department of Crop Production Ecology, Swedish University of Agricultural Sciences, 750 07 Uppsala, Sweden; 3 Department of Agronomy and Crop Science, Humboldt-University of Berlin, 14195 Berlin, Germany; 4 Institute of Crop Science and Resource Conservation, University of Bonn, 53115 Bonn, Germany; 5 Department of Agricultural Sciences, Viikki Plant Science Centre, University of Helsinki, 00014 Helsinki, Finland; 6 Crop and Soil Systems, Scotland’s Rural College, Aberdeen, AB21 9YA UK; 7 Institute for Resistance Research and Stress Tolerance, Julius Kuehn Institute, 18190 Sanitz, Germany

**Keywords:** Coefficient of variation, Pulses, Scaling, Taylor’s power law, Yield variability

## Abstract

Grain legumes produce high-quality protein for food and feed, and potentially contribute to sustainable cropping systems, but they are grown on only 1.5% of European arable land. Low temporal yield stability is one of the reasons held responsible for the low proportion of grain legumes, without sufficient quantitative evidence. The objective of this study was to compare the yield stability of grain legumes with other crop species in a northern European context and accounting for the effects of scale in the analysis and the data. To avoid aggregation biases in the yield data, we used data from long-term field experiments. The experiments included grain legumes (lupin, field pea, and faba bean), other broad-leaved crops, spring, and winter cereals. Experiments were conducted in the UK, Sweden, and Germany. To compare yield stability between grain legumes and other crops, we used a scale-adjusted yield stability indicator that accounts for the yield differences between crops following Taylor’s Power Law. Here, we show that temporal yield instability of grain legumes (30%) was higher than that of autumn-sown cereals (19%), but lower than that of other spring-sown broad-leaved crops (35%), and only slightly greater than spring-sown cereals (27%). With the scale-adjusted yield stability indicator, we estimated 21% higher yield stability for grain legumes compared to a standard stability measure. These novel findings demonstrate that grain legume yields are as reliable as those of other spring-sown crops in major production systems of northern Europe, which could influence the current negative perception on grain legume cultivation. Initiatives are still needed to improve the crops agronomy to provide higher and more stable yields in future.

## Introduction

**Fig. 1:**
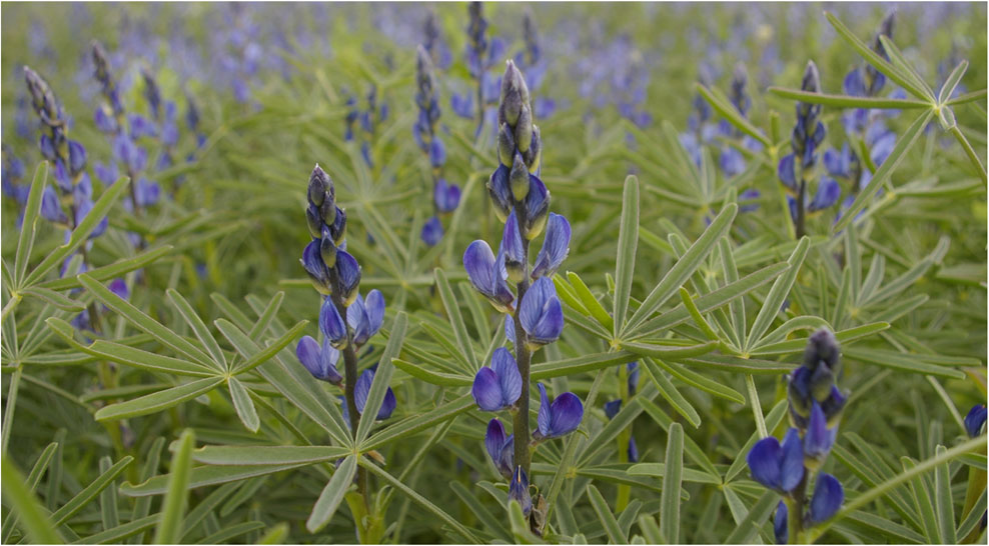
Narrow-leafed lupin (*Lupinus angustifolius* L.) grown in a long-term field experiment in northeastern Germany. Photo credit: Reckling/ZALF

Grain legumes produce high-quality protein for food and feed, and contribute to sustainable cropping systems by fixing nitrogen, increasing soil fertility and yields in subsequent crops, potentially reducing greenhouse gas emissions and supporting biodiversity (Watson et al. 2017) (Fig. 1). Grain legumes have also been shown to reduce trade-offs between economic and environmental impacts in various cropping systems across Europe (Reckling et al. 2016). Nevertheless, grain legumes were cultivated on only 1.5% of the arable land in Europe in 2014 (Watson et al. 2017). Research addressing the low adoption by farmers identified low productivity, low economic gains, insufficient economic valuation of external impacts, technological lock-in, and a low temporal yield stability as major constraints for the competitiveness of legumes with other crops (Zander et al. 2016; Magrini et al. 2016). Regarding yield stability, Hawtin and Hebblethwaite (1983), Peltonen-Sainio and Niemi (2012), and Cernay et al. (2015) all found lower yield stability in a range of grain legumes than in cereals.

A robust and scientifically sound analysis of temporal yield stability of grain legumes is relevant for the debate about legumes in the agricultural and policy sector, considering that it has been perceived as a major constraint to legume production by farmers, grower organizations, and experts from the European Innovation Partnership program (Zimmer et al. 2016; Von Richthofen et al. 2006; PGRO 2018; EIP-AGRI 2014). Nevertheless, this perception has not been convincingly tested, as available studies on legume yield stability have used average yield data from national statistics, which introduces a scale-dependent bias as crops, such as winter wheat, which are grown on larger areas and on better soils are compared with crops, such as narrow-leafed lupin, which are grown on much smaller areas and often on less fertile soils. At this scale of data aggregation, the processes affecting yield stability are averaged out, resulting in apparently higher stability than observed at the field level. This was illustrated by Popp et al. (2005) who showed that aggregated data under-estimated field-level yield risk in Canada. None of the above-mentioned studies tested the differences in yield stability between spring-sown and autumn-sown crops statistically, despite the fact that grain legumes are primarily spring-sown in Central and Northern Europe and should be compared with spring-sown cereals and spring-sown broad-leaved crops. Differences between broad-leaved crops and cereals have also been neglected in scientific literature up to now. The existing studies comparing cereals and legumes have also used relatively simple stability indicators such as the coefficient of variation (CV). Döring et al. (2015) highlighted the dangers of using stability indicators that are related to the mean yield and hence lead to an apparently poorer stability of crops with lower yields such as grain legumes compared to cereals.

The robust analysis of yield stability therefore requires field-level measurements to avoid scaling and aggregation biases and indicators that measure stability independent of the mean yield. Long-term field experiments offer yield data under relatively controlled conditions where all crops are grown on the same soil, under similar management, and at the same proportion of land over long time periods. Even though the existing long-term experiments do not all have the same design, and do not always include all crops that are currently the most economically relevant, they are ideal cases for studying temporal yield stability of crops, thanks to the controlled conditions and long time series available.

Taylor’s Power Law (TPL) can be used to derive a measure of stability that is independent of the mean. It describes the empirical relationship of the variance and the mean in a given dataset and states that the logarithm of the sample variance is a linear function of the logarithm of the sample mean across different subsets of data (Taylor 1961). It is one of the most widely confirmed empirical patterns in ecology (Cohen and Xu 2015), and Döring et al. (2015) showed that it also holds for several sets of crop yield data. To account for this systematic relationship, the residuals from the TPL regression can be used as a measure of yield stability that is independent of the mean yield (Reckling et al. 2015; Döring et al. 2015). This stability measure is called Power Law Residuals (POLAR). In this study, we apply and compare the standard CV with a new stability measure called “scale-adjusted coefficient of variation” (aCV) that combines the benefits of POLAR by removing dependence from the mean yield with the expression in intuitive units, i.e., as percentage of the mean (Döring and Reckling 2018). Quantification of grain legume yield stability at the field level, using a scale-adjusted stability measure, can then be used in the evaluation of the potential and limitations of grain legumes in European cropping systems.

The objective of this study is to assess whether yields of grain legumes are more or less stable than those of other crop species using field-level data from long-term field experiments from Northern Europe and by accounting for Taylor’s power law. We hypothesize that yields of grain legumes are less stable than those of other crop species, and that the novel scale-adjusted CV reduces the dependency of the standard CV on the mean yield.

## Materials and methods

In a first step, we tested the validity of TPL and the adjusted CV to analyze yield stability independent of the mean yield. Second, we applied the adjusted CV to compare yield stability of different crop groups across all experiments, and third, we investigated whether the general findings were also true for each of the experiments separately. Fourth, we quantified dry matter and protein yields, and finally (fifth), we discussed agronomic implications of our results.

### Dataset

For studying yield stability, long-term data from different sites and under controlled conditions were needed, which led to the choice of the experiments. Hence, five long-term field experiments conducted in the UK, Sweden, and Germany were chosen. These experiments were established at different times and with different purposes and vary in their design, length, and bio-physical characteristics. They provided 3768 site-year combinations. With regard to crop species and crop groups, datasets were balanced, i.e., at any one site, each crop species and crop group was grown every year, allowing analysis within sites and between sites. In all experiments, grain and tuber yields were harvested and moisture determined. For the analysis, fresh weights were converted into dry matter. To obtain protein yields, dry matter yields were converted into protein yields using standard conversion factors for crude protein from Feedipedia. All calculations were made on a 100% dry-matter basis.

Groups of crops were defined as “grain legumes” including faba bean, pea, and lupins (yellow and narrow-leafed); “cereals” including, spring barley, spring oat, spring wheat, winter barley, winter rye, winter spelt, winter triticale, and winter wheat; and “broad-leaved crops” including potato, sugar beet, and winter oilseed rape. “Spring-sown crops” were defined as potato, sugar beet, grain legumes, and spring cereals and “autumn-sown crops” as winter cereals and winter oilseed rape.

The experiments include a diversity of cropping systems to compare yield stability of different crop species. Sites differed in soil texture with clay content varying from 3 to 25%, annual precipitation from 545 to 667 mm, and annual mean temperature from 8.3 to 10.1 °C. Crop management differed in terms of rotations, organic vs. conventional management, crop species, cultivars, and fertilizer treatments. The main characteristics of the experiments are outlined below.

#### The Borgeby experiment R4–0002 at the Swedish University of Agricultural Sciences

The experiment is located at Borgeby in southern Sweden at 55°43.9′N and 13°2.0′E with an annual precipitation of 666 mm, an annual air temperature of 8.3 °C, and an oceanic climate. The soil is sandy with 11% clay and 63% sand and its pH is 6.0–6.3. It compares three cropping systems with differences in crop diversification and nitrogen fertilizer treatments (St-Martin et al. 2017). Yield data from 1960 to 2015 of winter wheat, sugar beet, spring barley, winter oilseed rape, spring wheat, and field pea were used for the analysis.

#### Agrometeorological field experiment at Humboldt-University of Berlin

The experiment is located in Berlin-Dahlem in northeastern Germany at 52°27.9′N and 13°17.9′E with an annual precipitation of 545 mm, an annual air temperature of 9.3 °C, and semi-continental climate characterized by occasional cold winters and hot summers (Chmielewski and Köhn 1999). The soil is heterogeneous and a silty sand with 3% clay and 73% sand, and its pH is 5.8. Yield data from 1953 to 2008 of yellow lupin, spring barley, sugar beet, spring oat, faba bean, winter rye, and potato were used for the analysis.

#### Crop rotation experiments (organic and conventional) at the Julius Kuehn Institute

The experiment is located in Groß Lüsewitz in northern Germany at 54°4.3′N and 12°20.1′E with an annual precipitation of 610 mm, an annual air temperature of 10.1 °C, and an oceanic climate. The soil is loamy sand with 10% clay and 65% sand and its pH is 5.8. Two experiments were conducted, one with conventional and one with organic management, comparing quality parameters of different cultivars. Yield data from 2003 to 2015 of potato, spring barley, spring wheat, narrow-leafed lupin, faba bean, field pea, winter barley, winter rye, winter spelt, winter triticale, and winter wheat were used for the analysis.

#### The Broadbalk experiment at Rothamsted Research

The experiment is located in Harpenden in southern England at 51°48.6′N and 0°21.4′W with an annual precipitation of 667 mm, an annual air temperature of 9.3 °C, and a temperate oceanic climate (Dyke et al. 1983). The soil is a clay loam to silty clay loam with 25% clay, 25% sand, and a pH of 7.0–7.5. For this study, the yield data from 1968 to 1978 of faba bean, winter wheat, and potato were used.

### Calculation of yield stability

In order to estimate the mean ($\widehat{\mu}$) and variance (${\widehat{\upsigma}}^2$) from several observations for the same experiment or treatment, the data were divided into subsets. Each series of data per experiment and treatment was divided into subsets of 8 years representing the maximum rotation length. The 8-year subsets resulted in a total of 471 observations containing information on the site, crop, grain, or tuber yield (Mg ha^−1^), protein yield (Mg ha^−1^), the mean ($\widehat{\mu}$), and variance (${\widehat{\upsigma}}^2$) of yield. The *n* pairs of means $\widehat{\mu}$_*i*_ and variances $\widehat{\upsigma}$_*i*_ (with index *i* from 1 to *n*) were used for calculating yield stability.

For the analysis of yield stability, a new version of the coefficient of variation was used, allowing adjusting for scaling effects of the mean yield. This stability measure is called adjusted coefficient of variation (aCV) and is based on Taylor’s Power Law (TPL), i.e., the linear relationship between log(${\widehat{\upsigma}}^2$) and log($\widehat{\mu}$) for the crop yield observations (Döring and Reckling 2018) and is a further development of the POLAR index (Döring et al. 2015).

For comparison with the adjusted coefficient of variation, the *standard* coefficient of variation CV_*i*_ was calculated as

1 $$ {\mathrm{CV}}_i=\frac{{\widehat{\upsigma}}_i}{{\widehat{\mu}}_i}\bullet 100\% $$

This CV assumes a linear rather than a log-log relationship between yield and variance. The adjustment of the coefficient of variation followed four steps. First, following TPL, a linear regression was calculated for log_10_ of the variance over the log_10_ of the mean of all crops. This is done following Döring et al. (2015) to obtain a linear regression for the whole dataset. With *v*_*i*_ = log(${\widehat{\upsigma}}^2$_*i*_) and *m*_*i*_ = log($\widehat{\mu}$_*i*_), the linear regression was *v* = *a* + *bm*. Second, the residuals *u*_*i*_ from this regression line, i.e., the POLAR, were calculated as

2 $$ {u}_i={v}_i-\left(a+b{m}_i\right) $$

Third, to account for the systematic relationship between the logarithm of the sample variance and the logarithm of the sample mean described by Taylor (1961), we adjusted the logarithm of the variance which was subsequently used for calculating the coefficient of variation. The adjusted logarithm of the variance ${\overset{\sim }{v}}_i$ is

3 $$ {\overset{\sim }{v}}_i=2{m}_i+\left(\mathrm{b}-2\right)\overline{m}+a+{u}_i $$

where $\overline{m}=\frac{1}{n}\sum {m}_i.$The fourth and final step was using the adjusted logarithm of the variance for calculating the adjusted coefficient of variation *aCV*_*i*_.

4 $$ {aCV}_i=\frac{\sqrt{g^{{\overset{\sim }{v}}_i}}}{{\widehat{\mu}}_i}\bullet 100\% $$

When the TPL regression slope *b* is < 2, the *standard* CV decreases non-linearly with increasing mean. In this case, *CV*_*i*_ = ${\widehat{\mu}}_i^{\frac{b}{2}-1}{g}^{\frac{a}{2}}\bullet 100\%$, where *g* is the basis of the logarithm (Döring et al. 2015). For adjusting the coefficient of variation, we removed the dependence of the CV from the mean by setting the slope *b* to 2, so that ${\widehat{\mu}}_i^{\frac{b}{2}-1}={{\widehat{\mu}}_i}^0$ = 1.

### Statistical analysis

All statistical analysis was performed with the R software version 3.3.1. Both, CV and aCV, were tested for normal distribution. For testing the relationship between the log(${\widehat{\upsigma}}^2$) and log($\widehat{\mu}$), the CV and the logarithm of the mean yield, and the aCV and the logarithm of the mean yield, a linear model was applied with the *lm* function in R.

For testing significant differences in dry matter yield, protein yield, aCV, and CV for each site separately, an ANOVA and the Tukey’s HSD test were used (pairwise comparisons of multiple means).

Significant differences for dry matter yield, protein yield, aCV, and CV between groups of crops, grain legume species, and the ranking of crops at all sites were tested using a linear mixed effects model with the *lme* function in R, using site as a random factor and crop group or species as a fixed factor.

#### Data availability

The datasets used in this study were sourced from the Swedish University of Agricultural Sciences, Rothamsted Research, Humboldt-University of Berlin and Julius Kuehn Institute under license for the current study. The datasets are not publicly available but may be obtained from the authors upon reasonable request and with the permission of the institutions mentioned above.

## Results and discussion

### Analysis of yield stability independent of the mean yield

**Fig. 2:**
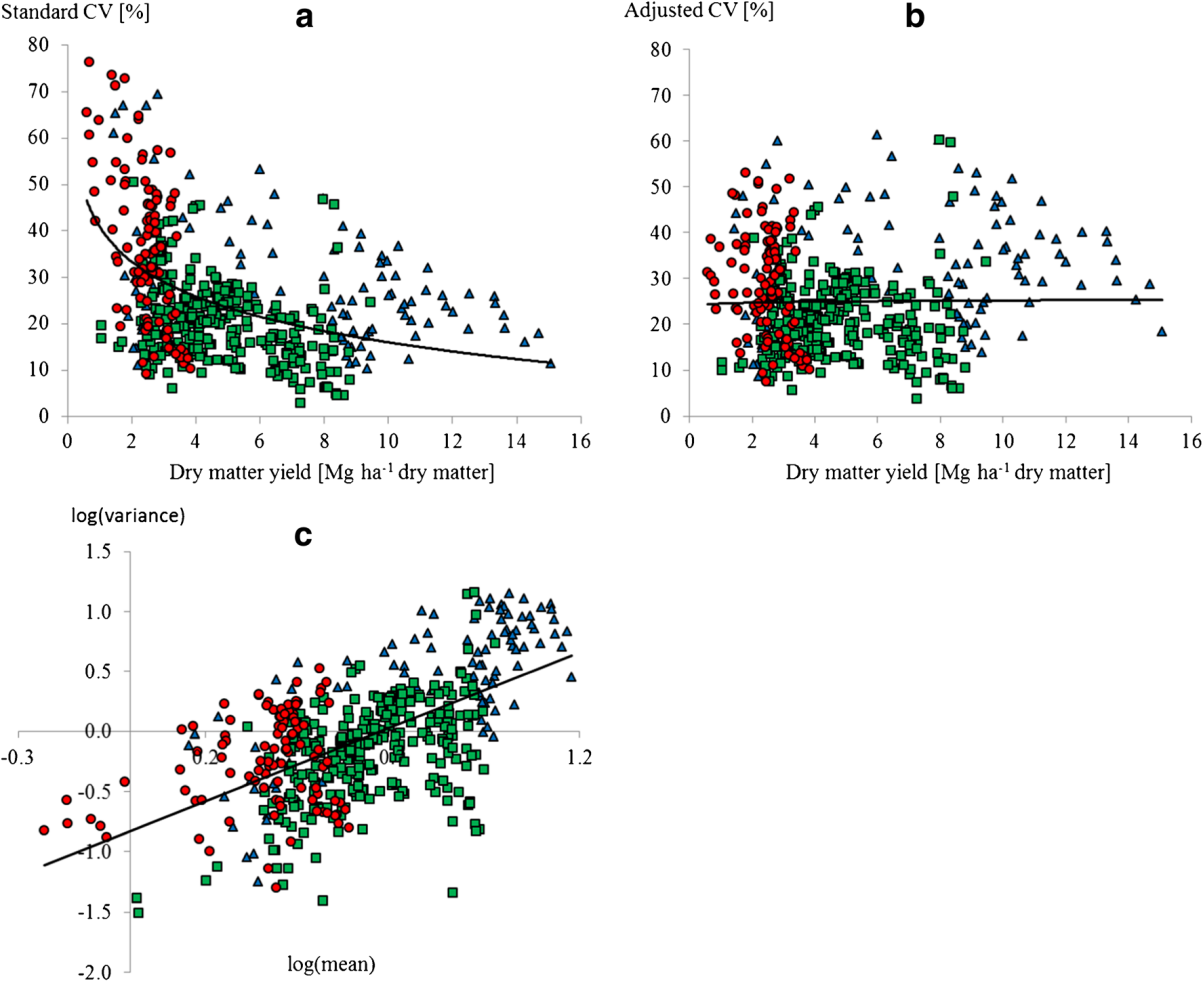
Relationship between dry matter yield and **a** the standard coefficient of variation (CV) and **b** the adjusted CV, and **c** between the logarithm of the variance against the logarithm of the mean. Each data point represents the mean and variance of an 8-year period from long-term experiments for grain legumes (*n* = 100, red circles), other broad-leaved crops (*n* = 96, blue triangles), and cereals (*n* = 275, green squares). The relationship is shown with a logarithmic regression line (**a**, **b**) and a linear regression line (**c**) over all groups of crops (*n* = 471)

The CV was negatively correlated with the dry matter yield (intercept = 40.82 ± 1.48 and slope = − 10.77 ± 0.97, df = 469, adjusted R^2^ = 0.206, *P* < 0.001) (Fig. 2a). Grain legumes had the lowest yields, < 4 Mg ha^−1^, and the largest range of CV values from 9 to 76%.

When TPL was applied to remove the dependence of the CV on the mean yield, the logarithm of the variance increased linearly and significantly against the logarithm of the corresponding mean from the 471 observations (intercept = − 0.83 ± 0.05 and slope = 1.24 ± 0.08, df = 469, adjusted *R*^2^ = 0.357, *P* < 0.001) (Fig. 2c).

In contrast to the dependence of the CV on yield, the aCV obtained by applying TPL was independent of the yield (Fig. 2b), showing that this new indicator can be used to determine yield stability independent of the mean. Adjusting the CV for yield dependence had different effects on the apparent yield stability of different groups of crops. For grain legumes, the estimated yield instability was lower using the aCV (30%) than the CV (38%) whereas for spring-sown broad-leaved crops, the yield instability was higher with the aCV (35%) than with the CV (27%) (Table 1). The two methods gave a similar estimate for yield instability of spring-sown (aCV 27% and CV 28%) and autumn-sown cereals (both 19%) (Table 1). As the aCV measured yield instability independent of the mean yield, it was used for all subsequent assessments in this study.

**Table 1:** Comparisons in yield stability estimated with the adjusted coefficient of variation (aCV) and the coefficient of variation (CV) between different groups of crops

Comparison^a^	aCV (%)					CV (%)				
	Mean	Std. error	DF	t-value	*p* value	Mean	Std. error	DF	t-value	*p* value
*Spring-sown crops with autumn-sown crops*										
s	30	2.3				31	2.1			
s:a	− 10	0.9	466	− 10.58	< 0.001	− 11	1.2	466	− 9.09	< 0.001
*Grain legumes with cereals and broad-leaved crops*										
sGL	30	1.9				39	2.0			
sGL:s + aCR	− 8	1.2	465	− 6.38	< 0.001	− 16	1.4	465	− 11.86	< 0.001
sGL:s + aBL	4	1.5	465	2.50	0.013	− 10	1.7	465	− 5.82	< 0.001
*Grain legumes with non-legume spring-sown and autumn-sown crops*										
sGL	30	2.4				39	2.1			
sGL:sNL	0	1.2	465	0.20	0.845	− 11	1.4	465	− 7.91	< 0.001
sGL:aNL	− 10	1.2	465	− 7.90	< 0.001	− 18	1.4	465	− 12.49	< 0.001
*Grain legumes with other spring-sown and autumn-sown crops*										
sGL	30	2.1				38	2.2			
sGL:sCR	−3	1.3	463	− 2.04	0.042	− 10	1.5	463	− 6.81	< 0.001
sGL:aCR	−11	1.2	463	− 9.10	< 0.001	− 20	1.4	463	− 13.97	< 0.001
sGL:sBL	5	1.4	463	3.60	< 0.001	− 11	1.7	463	− 6.60	< 0.001
sGL:aBL	−1	2.4	463	− 0.53	0.594	− 2	2.8	463	− 0.76	0.445
*Lupins with faba bean and pea*										
sLU	23	3.2				34	6.1			
sLU:sFB	14	2.4	94	6.05	<0.001	15	3.3	94	4.37	< 0.001
sLU:sPE	7	2.8	94	2.54	0.013	7	3.9	94	1.87	0.064

s spring-sown crops, a autumn-sown crops, GL grain legumes, CR cereals, BL broad-leaved crops, NL non-legume crops, LU lupins, FB faba bean, PE pea

^a^Statistics were performed using a linear mixed effects model, using site as a random factor and crop group or species as a fixed factor

Our results showed that TPL, a widely verified quantitative pattern in ecology (Cohen and Xu 2015), can be used effectively to compare yield stability between different crop species grown in long-term experiments. Using the log-linear relationship between yield and variance in an aCV instead of the linear relationship assumed in the standard CV changed the ranking of yield stabilities of crop groups (see the size of the sample for each crop group in Fig. 2).

### Yield stability among groups of crops across all experiments

**Fig. 3:**
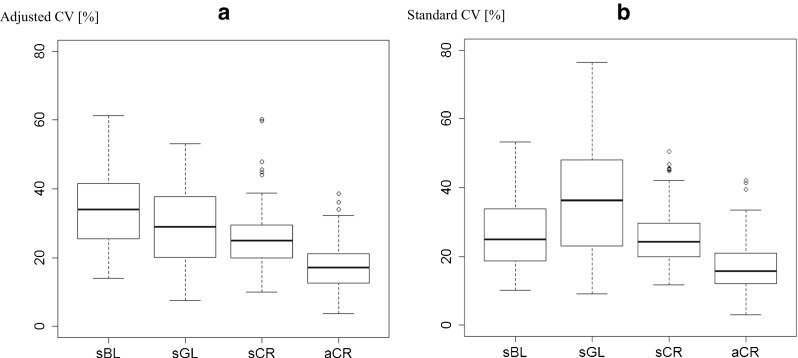
Yield stability of different crop groups, **a** estimated with the adjusted coefficient of variation (CV) and **b** with the standard CV. Comparison between spring-sown broad-leaved crops (sBL) (*n* = 75), spring-sown grain legumes (sGL) (n = 100), spring-sown cereals (sCR) (*n* = 117), and autumn-sown cereals (aCR) (*n* = 158). In each boxplot, the median is the black bar, the box covers the interquartile range, the whiskers cover the entire range of data, and circles indicate potential outliers

Yield stabilities estimated with the aCV were 16% points, 11% points, and 8% points higher for autumn-sown cereals than for spring-sown broad-leaved crops, spring-sown grain legumes, and spring-sown cereals, respectively, across all experiments and species (Fig. 3). Grain legume yields were 5% points more stable than those of other spring-sown broad-leaved crops (*P* < 0.001) and 3% points less stable than spring-sown cereals (*P* < 0.05) (Table 1). Overall, yields of autumn-sown crops were 10% points more stable than spring-sown crops (*P* < 0.001) (Table 1) and the grain legume yields were more stable than yields of broad-leaved crops (*P* < 0.05). There was no difference in yield stability between grain legumes (all spring sown) and all non-legume spring-sown crops (spring-sown cereals and broad-leaved crops) using the aCV (Table 1).

### Yield stability among crop species within each experiment

The analysis of yield stability within each of the five experiments supported the general findings with grain legumes being similar stable as other spring-sown crops and broad-leaved crops. Lupin (yellow and narrow-leafed) yields were on average 14% points and 7% points more stable than those of faba bean and field pea, respectively (Table 1). There were differences across sites, with high stability of narrow-leafed lupin at Groß Lüsewitz and a low stability for yellow lupin at Dahlem (Table 2). Yield of faba bean was unstable at all sites and that of field pea was least stable at Borgeby, but as stable as cereal yields in the organic system at Groß Lüsewitz (Table 2). Organic management studied at Groß Lüsewitz reduced mean yields compared to the conventional system (Table 2), but yield stabilities of field pea and faba bean were greater in the organic system than in the conventional. Potato and faba bean were among the least stable and winter wheat and winter rye among the most stable crops across all experiments (Table 2).

**Table 2:** Adjusted coefficient of variation (aCV), coefficient of variation (CV), dry matter yield, and protein yield per crop species and site

Site	Crop group	Species	n	aCV (%)		CV (%)		Dry matter yield (Mg ha^−1^)		Protein yield (Mg ha^−1^)	
Borgeby	Grain legume	Field pea	14	36	c	50	c	1.8	a	0.44	a
	Broad-leaved crop	Sugar beet	21	23	ab	17	a	10.0	d	0.78	b
		Winter oilseed rape	21	27	abc	34	b	2.3	a	0.48	a
	Cereals	Spring barley	28	29	bc	25	ab	5.8	c	0.68	b
		Spring wheat	21	22	ab	23	a	3.6	b	0.45	a
		Winter wheat	35	19	a	18	a	5.2	c	0.65	b
Berlin-Dahlem	Grain legume	Yellow lupin	7	30	bc	55	b	0.9	a	0.31	a
		Faba bean	7	36	c	47	b	2.2	b	0.62	b
	Broad-leaved crop	Potato	7	35	c	26	a	9.0	d	1.15	d
		Sugar beet	7	30	bc	20	a	10.9	e	0.85	c
	Cereals	Spring barley	7	20	ab	23	a	3.0	bc	0.35	a
		Spring oat	7	25	abc	29	a	2.6	bc	0.30	a
		Winter rye	7	15	a	16	a	3.7	c	0.62	b
Groß Lüsewitz, conventional	Grain legume	Narrow-leafed lupin	10	16	b	17	bcd	3.4	ab	1.15	e
		Faba bean	10	42	e	48	g	3.0	a	0.86	cd
		Field pea	8	32	cd	38	f	2.6	a	0.62	ab
	Broad-leaved crop	Potato	12	35	de	24	sde	11.6	g	1.48	f
	Cereals	Spring barley	15	27	c	25	de	5.3	c	0.63	ab
		Spring wheat	12	27	c	27	e	4.2	b	0.53	a
		Winter barley	13	19	b	16	b	6.6	de	0.78	c
		Winter rye	11	16	b	12	ab	7.6	ef	1.28	e
		Winter spelt	6	18	b	16	bc	5.8	cd	0.73	bc
		Winter triticale	11	19	b	15	b	7.9	f	0.99	d
		Winter wheat	9	8	a	6	a	7.9	f	0.99	d
Groß Lüsewitz, organic	Grain legume	Narrow-leafed lupin	10	16	a	17	a	3.2	cd	1.07	f
		Faba bean	10	25	bc	32	b	2.1	a	0.60	d
		Field pea	8	15	a	19	a	2.5	ab	0.59	cd
	Broad-leaved crop	Potato	12	41	d	37	b	5.6	g	0.71	e
	Cereals	Spring barley	15	29	c	32	b	3.0	bcd	0.36	a
		Spring wheat	12	18	ab	22	a	2.5	ab	0.31	a
		Winter barley	13	19	ab	21	a	3.5	de	0.41	ab
		Winter rye	11	17	ab	17	a	4.3	f	0.72	e
		Winter spelt	6	12	a	14	a	3.0	bcd	0.37	ab
		Winter triticale	11	20	ab	21	a	7.9	ef	0.99	bc
		Winter wheat	9	12	a	14	a	2.7	abc	0.34	a
Harpenden	Grain legume	Faba bean	16	35	b	43	c	2.5	a	0.72	a
	Broad-leaved crop	Potato	16	46	c	38	b	7.6	c	0.97	b
	Cereals	Winter wheat	16	20	a	20	a	4.4	b	0.55	a

Letters indicate significant difference per site at *P* < 0.05 using an ANOVA and the Tukey’s HSD test for pairwise comparisons of multiple means

*n* no. of 8-year periods

The lack of evidence for a higher instability for grain legumes than for other spring-sown crops leads us to reject our hypothesis about an inherent instability of grain legume yields in long-term experiments from northern Europe. This conclusion was supported by the analysis of all experiments together (Table 1) and the analysis for each experiment separately (Table 2). Exceptions were lupins that were more stable than spring-sown cereals and sugar beet that were more stable than pea and faba bean but less stable than cereals and lupins.

These novel findings contrast with previous research (Hawtin and Hebblethwaite 1983; Cernay et al. 2015; Peltonen-Sainio and Niemi 2012) and with farmers’ and experts’ perceptions (Von Richthofen et al. 2006; Zimmer et al. 2016; PGRO 2018; EIP-AGRI 2014). First, farmers may perceive grain legumes to be less stable because of relatively low prices and missing value chains (Preissel et al. 2017) and because of agronomic constraints with pests, diseases, and weeds. These constraints might be less visible in long-term experiments that were designed according to good agricultural practices, which not all existing cropping systems are. Second, all previous studies have used indicators that do not correct for the scaling effects of the log-linear association of the variance with the mean yield, which our results show over-emphasizes variation in low-yielding crops. Third, when data are aggregated at the national level in official statistics, there is a tendency that the apparent yield stability increases with the size of the harvested area (Cernay et al. 2015). We propose that the latter two aspects result in lower apparent stability for grain legumes with generally lower yields than many other crops (Table 2) and that are grown on < 2% of the arable land in Europe. Since the indicator for estimating yield stability was scale-adjusted by using the aCV in the present study (Fig. 2b), and field-level data from long-term experiments were used, where all crops are grown in the same proportion and on the same plot size, we effectively dealt with the inappropriate effects of scale on measures of stability that have been used in earlier studies. We removed the significant relationship between yield and the standard CV (Fig. 2a) that explains higher stability of high-yielding crops and lower stability of low-yielding crops, supporting the initial hypothesis that scale adjustment was important. Other existing variance and regression-based indicators to analyze yield stability (Piepho 1998) do not account for this dependence on mean yield. Thus, the aCV is an important addition to the set of stability indicators available in plant sciences.

The importance of using field-level data in contrast to aggregated data where variability is averaged out is obvious from the present study. The average CV value of 26% from the long-term experiments was twice as great as the average CV of 13% from the studies with national aggregated yield data (Cernay et al. 2015; Peltonen-Sainio and Niemi 2012). Long-term experiments have not been previously used sufficiently to assess yield stability, but since there are several hundreds of experiments available (620 are listed in a global assessment by Debreczeni and Körschens (2003) alone), this resource could be exploited more effectively in the future. The three problems with long-term experiments are that first, not all crops of current economic importance are grown; second, many are unbalanced so that not all crops are grown in the same year; and third, the small plot size that can result in lower stability of crops that are disadvantaged in small plots. The latter is due to yield losses because of biotic stresses, as is the case for grain legumes. Kravchenko et al. (2017) found that the yield gap between experiments with small plots and field-scale experiments with large fields was more pronounced for soybean and maize than for wheat. This indicates that legumes would come out even better in comparison to other crop groups if the plot size were larger than is the case in most long-term experiments.

### Dry matter and protein yield

Grain yields of grain legumes were on average 50 and 69% lower than those of cereals and broad-leaved crops, respectively (Table 2). In some of the sites, grain legume yields were not significantly different from those of other crops, e.g., field pea and winter oilseed rape at Borgeby, and narrow-leafed lupin and most cereals in the organic system at Groß Lüsewitz (Table 2). Narrow-leafed lupin had on average 11 and 25% higher yields than faba bean and field pea. Faba bean yields averaged 11% higher than pea. Yellow lupin had the lowest grain yields of all crops at Berlin-Dahlem (Table 2).

Protein yields were 16% higher for grain legumes than for cereals (Table 2) due to the higher protein content in the legume (24–39%) than in the cereal (11–17%). Broad-leaved crops had the highest protein yield due to their, on average, relatively high yields and protein contents, even if the differences in both yield and protein content were large among species, i.e., the protein content was 8% for sugar beet, 13% for potato, and 21% for winter oilseed rape. Narrow-leafed lupin had higher protein yields than faba bean and pea (Table 2) because of combined higher yield and protein content at the Groß Lüsewitz site. Yellow lupin had the highest protein content (39%) but the lowest protein yield of all grain legumes due to its low grain yield (Table 2).

Besides an increase in the area cultivated with grain legumes, a yield increase is needed for grain legumes in northern Europe to effectively reduce the dependency on imports (Zander et al. 2016). Among grain legumes, narrow-leafed lupin is considered as a potentially important protein crop in Europe (Lucas et al. 2015) and our results support this potential with high protein yields and high yield stability. However, the production and use of narrow-leafed lupin is constrained by often high alkaloid content in the seed, poor growth on alkaline soils and high pest, disease, and weed pressure (Jansen et al. 2015).

### Agronomic implications

We found that yields of grain legumes were more stable than those of other spring-sown broad-leaved crops, i.e., potato and sugar beet, which might be because these crops are known to be poor at adapting to environmental stresses. Potato was the major broad-leaved crop in this study with the lowest yield stability. It is affected by different aphid species, potato viruses, and late-blight (*Phytophthora infestans* (Mont.) de Bary) and is sensitive to drought, low temperatures, solar radiation, and evapotranspiration during tuber formation (Peltonen-Sainio et al. 2010). Sugar beet yield was more stable than that of the other broad-leaved crops and is dependent on sufficient water supply, and both low temperatures and reduced evapotranspiration lead to lower yield formation (Peltonen-Sainio et al. 2010).

The lower yield stability in grain legumes than in autumn-sown cereals can be attributed to several factors. First, all grain legumes investigated were spring-sown, and spring-sown crop yields were generally more unstable than those of autumn-sown crops (Table 1). In northern Europe, spring-sown crops can be constrained by water deficits during crop establishment and subsequent growth stages, whereas winter crops are established in autumn and regrow quickly after winter without any delays due to soil tillage and seedbed preparation that can also reduce soil moisture. Autumn-sown crops often have deeper root systems that allow access to water in deeper soil layers (Thorup-Kristensen et al. 2009), and they mature earlier, i.e., a larger part of their growth is in cool temperatures with higher water availability. There was no data available that allowed for the analysis of autumn-sown grain legumes, but on the basis of the available comparisons in other crop groups, higher stability can be expected in regions with mild winters such as the UK and western France. In northern Europe with cold winters, autumn-sown grain legumes are likely to fail so they are not grown as winter crops. Second, grain legumes have an indeterminate growth habit that allows the crop to respond to good conditions such as high water availability and adequate temperature or to stop growing and reproducing in poor conditions (Stoddard et al. 2006), whereas cereals can compensate in conditions of sufficient or insufficient water and nutrient supply through tillering and flower initiation, and the corresponding reductions. Third, symbiotic nitrogen fixation affects yield and can be reduced or fail in poor conditions resulting in greater yield instability. Furthermore, protein is a more energy-rich product than carbohydrate, so the high protein content of legumes may represent an intrinsic yield penalty. Finally, the investment in breeding of any major cereal for yield, disease resistance, and stress tolerance greatly exceeds that in grain legumes, except soybean (Magrini et al. 2016), which could influence the hardiness of plants when confronted with stresses. There is also an intrinsic limitation of legume crop yields due to their costly seed composition. Using theoretical calculations with legumes, Munier-Jolain and Salon (2005) demonstrated a negative relationship between the carbon cost of seed production and yield.

Many factors affecting yield stability in grain legumes can be managed by breeding or agronomy, especially well-designed rotations. Each species has different tolerances to abiotic stresses (Stoddard et al. 2006), is affected by different diseases (Watson et al. 2017), and shows wide intraspecific variation in stress response, but in this study, yield stability differences were examined only at the species level. The greater yield stability of lupin in the three German sites of the present study may be due to the greater tolerance of dry conditions and sandy soils attributed to both narrow-leafed lupin and yellow lupin in comparison with pea and faba bean. Lupin was also less affected by pests and disease except for the leaf weevil of the genus *Sitona.* In pea, root rot caused by *Aphanomyces euteiches* Drechsler and infestation by pea aphid *Acyrthosiphon pisum* Harris reduce yields significantly. In faba bean and other grain legumes, pathogenic fungi, such as different species of *Ascochyta*, *Botrytis*, and *Colletotrichum*, can cause crop failure in susceptible cultivars, so resistance breeding is a priority. Grain legumes are more susceptible to competition from weeds than cereals, because they are poor competitors for nutrients, establish slowly, and are susceptible to lodging that can open up for weeds.

## Conclusion

We conclude that yields of grain legumes are not inherently less stable than those of other spring crops in long-term experiments in northern Europe, as has been found in previous research using national yield data. The novel scale-adjusted aCV indicator removes the dependency of the standard CV on the mean yield and is a powerful tool to quantify yield stability of different crop species or cropping systems with large differences in mean yields. We highlight that care is needed when choosing data and methods to quantify yield stability and show the benefits of using long-term experiments and a scale-adjusted yield stability measure. Although our findings could change the current negative perception on grain legume cultivation in northern Europe, making them an effective option to increase the sustainability of cropping systems, initiatives are needed to improve the crops’ agronomy.
